# Non-Invasive Fetal Monitoring: A Maternal Surface ECG Electrode Placement-Based Novel Approach for Optimization of Adaptive Filter Control Parameters Using the LMS and RLS Algorithms

**DOI:** 10.3390/s17051154

**Published:** 2017-05-19

**Authors:** Radek Martinek, Radana Kahankova, Homer Nazeran, Jaromir Konecny, Janusz Jezewski, Petr Janku, Petr Bilik, Jan Zidek, Jan Nedoma, Marcel Fajkus

**Affiliations:** 1 Department of Cybernetics and Biomedical Engineering, Faculty of Electrical Engineering and Computer Science, VSB-Technical University of Ostrava, 17 Listopadu 15, 70833 Ostrava, Czech Republic; radana.kahankova@vsb.cz (R.K.); jaromir.konecny@vsb.cz (J.K.); petr.bilik@vsb.cz (P.B.); jan.zidek@vsb.cz (J.Z.); 2 Department of Electrical and Computer Engineering, University of Texas El Paso, 500 W University Ave, El Paso, TX 79968, USA; hnazeran@utep.edu; 3 Institute of Medical Technology and Equipment ITAM, 118 Roosevelt Str., 41-800 Zabrze, Poland; jezewski@itam.zabrze.pl; 4 Department of Obstetrics and Gynecology, Masaryk University and University Hospital Brno, Jihlavska 20, 625 00 Brno, Czech Republic; janku.petr@fnbrno.cz; 5 Department of Telecommunications, Faculty of Electrical Engineering and Computer Science, VSB-Technical University of Ostrava, 17 Listopadu 15, 70833 Ostrava, Czech Republic; jan.nedoma@vsb.cz (J.N.); marcel.fajkus@vsb.cz (M.F.)

**Keywords:** fetal ECG, adaptive filtering, Least Mean Squares (LMS) algorithm, Recursive Least Squares (RLS) algorithm

## Abstract

This paper is focused on the design, implementation and verification of a novel method for the optimization of the control parameters (such as step size μ and filter order *N*) of LMS and RLS adaptive filters used for noninvasive fetal monitoring. The optimization algorithm is driven by considering the ECG electrode positions on the maternal body surface in improving the performance of these adaptive filters. The main criterion for optimal parameter selection was the Signal-to-Noise Ratio (SNR). We conducted experiments using signals supplied by the latest version of our LabVIEW-Based Multi-Channel Non-Invasive Abdominal Maternal-Fetal Electrocardiogram Signal Generator, which provides the flexibility and capability of modeling the principal distribution of maternal/fetal ECGs in the human body. Our novel algorithm enabled us to find the optimal settings of the adaptive filters based on maternal surface ECG electrode placements. The experimental results further confirmed the theoretical assumption that the optimal settings of these adaptive filters are dependent on the ECG electrode positions on the maternal body, and therefore, we were able to achieve far better results than without the use of optimization. These improvements in turn could lead to a more accurate detection of fetal hypoxia. Consequently, our approach could offer the potential to be used in clinical practice to establish recommendations for standard electrode placement and find the optimal adaptive filter settings for extracting high quality fetal ECG signals for further processing. Ultimately, diagnostic-grade fetal ECG signals would ensure the reliable detection of fetal hypoxia.

## 1. Introduction

Currently, fetal electrocardiography (fECG) seems to offer the most promising method to detect fetal hypoxia [1,2,3]. Fetal hypoxia is a pathological state that occurs when the fetus is deprived of an adequate supply of oxygen. It can be caused by a number of reasons such as umbilical cord prolapses, cord occlusion or cord thrombosis, placental infarction and others. Although it was proposed several decades ago [4,5], its potential utility is not fully realized yet. This is mainly due to limitations in the reliability and accuracy of abdominally-extracted fECG signals [6].

The other promising methods are fetal phonography (fPCG) [7,8,9] and fetal magnetocardiography (fMCG) [10,11,12]. While fECG is based on recording the electrical activity of the fetal heart, fPCG deals with recording its mechanical (acoustical) activity, and the fMCG is a method that registers the associated magnetic fields of the fetal heart produced as a consequence of its electrical activity [8,9,13,14].

In today’s clinical practice, ultrasound technology is widely used for fetal heart rate (fHR) [15,16,17] detection, as it is both economical and simple to use. However, this approach has limitations due to the inherent characteristics of the ultrasound method, such as reliability, accuracy, fetal or maternal movement influences, as well as the impact of the maternal heart rate (mHR) on the fHR. Moreover, this method only produces an average heart rate value and cannot be used for monitoring the fetal heart’s beat-to-beat variability, as well as its fECG signal morphology analysis. In addition, the effect of ultrasound on the fetus is not clearly understood, and consequently, it cannot be recommended for long-term fetal monitoring [18].

Efforts to minimize the adverse impact of ultrasound factors on signal quality lead to limitations in mobility and comfort level of the patient. Consequently, the position of the ultrasound transducers must frequently be adjusted, which is time-consuming and places an extra burden on the clinical staff. Moreover, the quality of ultrasound Doppler-based recordings [19,20,21] is also affected by the high maternal Body Mass Index (BMI), which has been increasing in recent years [22,23]. During labor and delivery, an invasive method of fetal heart monitoring is used to ensure the more accurate detection of fetal hypoxia [24]. However, it carries the risk of infection and trauma for the mother and fetus alike and therefore cannot be used before cervical dilatation has occurred.

Current trends in this research area are gradually favoring the use of transabdominal ECG in clinical practice [21,25,26,27,28,29,30]. With this approach, since the ECG signals rather than the heart movements data are analyzed, it should be possible, in principle, to produce an accurate representation of the heart rate, as well as its variability. A number of research efforts show that the abdominal ECG approach compares well in accuracy and reliability with the ultrasound technique or even exceeds it [6,21,24].

As the underlying physical principle used for abdominal ECG monitoring is not affected by the amount of maternal adipose tissue (body fat), the quality of the measured signal is quite high; therefore, abdominal electrocardiography is the only technique that potentially makes long-term ambulatory fetal monitoring possible. In addition, the recent availability of wireless fHR-monitoring systems [31] substantiates the fact that with using such technologies, it is now possible for pregnant women to move freely during their early stages of labor.

Compared to its invasive transvaginal counterpart, transabdominal ECG is limited, as it only provides information about the fetal heart rate (fHR) and not its signal morphology. This is mainly due to the fact that the desired fECG signal (in comparison with interfering influences) is small in amplitude. When invasively recorded, the fECG signal is measured directly from the head of the unborn child via a scalp electrode secured transvaginally to the fetal fontanel. The transvaginal method, even though unpleasant and risky, produces a more accurate fECG signal compared to its noninvasive alternative.

In contrast, the noninvasively recorded abdominal ECG (aECG) signal is formed by the summation of different influences that overlaps the fECG signal during its propagation from the fetal heart to the maternal abdominal surface electrodes, with the major contribution coming from the maternal heart (mECG). The mECG signal overlaps with the fECG signal not only in time, but also in the frequency domain. Its bio-source (maternal heart) is large compared to the bio-source (fetal heart) of the desired signal. As such, the fECG signal is hardly noticeable in the maternal abdominal ECG recordings, and without proper signal processing, it is not possible to extract useful information from fECG signals. However, for the reasons mentioned above, the extraction of fECG signals from abdominal ECG is a very difficult signal processing task, and classical linear FIR filters have not been able to tackle this problem.

Fortunately, there are many nonlinear signal processing methods that have been used for fECG signal extraction. These methods can be divided into two general categories: (1) non-adaptive; and (2) adaptive. The difference lies in the inability or ability of a system to adapt to unexpected circumstances. Non-adaptive methodologies include: Wavelet Transforms (WTs) [32,33,34], correlation [35], signal subtraction [36], Single Value Decomposition (SVD) [37], Independent Component Analysis (ICA) [38], Blind Subspace Separation (BSS) [39] and signal averaging [40] techniques. Non-adaptive techniques are time-invariant in nature, which means that they are less effective in reducing the mECG signal, which overlaps the desired fECG signal in the time and frequency domains, than the adaptive methods. Several adaptive filtering approaches for mECG signal cancellation from the aECG signals and consequent fECG signal extraction have been used. Adaptive filters can be trained to extract the fetal QRScomplexes directly or to estimate and remove the mECG signal using reference maternal ECG channels. The reference mECG signal can be recorded from maternal thoracic electrodes or reconstructed from several abdominal channels that are linearly independent.

In turn, adaptive signal processing methods can be linear or non-linear. Linear adaptive methods used for fECG extraction include algorithms such as Least Mean Squares (LMS) [41,42], Recursive Least Squares (RLS) [41,42], comb filtering [43], adaptive Volterra filtering [44], Kalman filter [45,46] or Adaptive Linear Networks (ADALINE) [47]. Non-linear techniques are based on artificial intelligence and include fuzzy inference systems [48], genetic algorithms and Bayesian adaptive filtering frameworks [49,50]. In this paper, we focus on the design, implementation, testing and validation of adaptive filters using the LMS and RLS algorithms. However, in the future, we intend to extend this work and explore the utility of other adaptive systems in non-invasive fetal monitoring.

Generally speaking, the first step in using adaptive filtering methods is to focus on control parameter settings. However, the parameters of adaptive filters are often chosen based on previous knowledge and/or experience. Here, we have explored the influence of the adaptive filter control parameters (such as step size μ and filter order *N*) on the quality of the fECG signal and evaluated the performance of the filters mainly by using the Signal-to-Noise Ratio (SNR). As our approach is dependent on the placement of the maternal electrodes, it enables users to identify and map those anatomical positions (the so-called “operating areas”) for which adaptive filters produce the best outcomes. Using this information, it is possible to establish new recommendations for adaptive filter settings and enable users to achieve the best possible results. In summary, with our approach, it is possible to modify the adaptive filter settings to extract the best quality fECG signals based on changes in the electrode placements or anatomical differences among pregnant women.

It should be emphasized that to find the optimal adaptive filter parameters, we need to use synthetic data to generate the fetal signal (as noninvasive recording of such data is not possible), which is crucial for the determination of the quality measures of the extracted signal such as the SNR, $S^{+}$ (Sensitivity) and PPV (Positive Predictive Value) [51,52]. Moreover, by using synthetic data, we are able to model the fetal hypoxic conditions and determine whether the extracted fECG signals allow us to successfully determine fHR, as well as T/QRS values and, furthermore, reliably construct the fetal Heart Rate Variability (HRV) signal. Therefore, the major aim of this paper is to verify whether or not optimal adaptive filter settings depend on abdominal electrode positions. This is of significant research interest as, to the best of our knowledge, there is no research in the current literature that has made use of adaptive filtering algorithms to address this problem [46,53,54,55,56,57,58]. To develop our adaptive filter optimization algorithms reported here, we used our Novel Synthetic Maternal/Fetal ECG Signal Generator [59,60,61] to supply the necessary synthetic aECG signals.

## 2. State of the Art

Recent research efforts on the topic of fECG signal extraction have increased considerably [62,63,64]. A number of authors have used a rich variety of methods based on different principles for this purpose, as was mentioned in the previous section. For those cases in which only abdominal electrodes have been used to acquire the aECG signals, the fECG signals were extracted directly by means of linear [32,33,34,35,36,37,38,39,40,53,65,66,67,68,69,70,71,72] or nonlinear (for example, [73,74]) decomposition techniques [75,76].

In contrast, here, we pay special attention to the use of adaptive filtering methods [41,42,43,44,45,46,47,48,49,50,77,78,79,80,81] and their optimization, while acknowledging the disadvantage that such techniques have in requiring additional maternal thoracic electrodes and the associated lead wires to acquire the reference maternal ECG signal. Even though the reference maternal thoracic signal can be estimated from linearly independent abdominal ECG signals to remove the need for extra electrodes and wires, for more accurate results, it is preferable to directly record the reference maternal signal [14]. Our objective here is to show that optimized adaptive methods have the potential to be used not only for fHR detection, but also for the further detailed fECG signal wave shape extraction and morphology analysis.

Adaptive methods differ from one another. However, they have one aspect in common, and that is the need for setting their control parameters. In fetal electrocardiography, this is indeed a very challenging task as the optimal value of these parameters is based on many factors, such as fetal position, maternal electrode placement, stage of the pregnancy, and so on. Moreover, the parameter setting is individual for each patient, and it changes during the pregnancy. Therefore, to achieve good results, it is essential to dedicate comprehensive efforts to this topic.

In general adaptive filtering applications, the selection of filter parameters is relatively straightforward, intuitive and mostly based on previous experiences or the filtered signal quality. Similarly, in electrocardiography, electrode placement for the acquisition of good quality signals is well defined. For example, in the case of the clinical 12-lead electrocardiography, there is a standardized placement for each electrode, which is nonexistent for noninvasive fetal ECG monitoring. Some authors use a four-lead system; some prefer using more leads. The location of the electrodes also differs [75]. For these reasons, we found it is essential to investigate the influence of these factors on fetal ECG monitoring and come up with evidence-based recommendations regarding maternal electrode placement and the parameter settings used in adaptive filtering of the maternal abdominal ECG based on our findings.

In addition, in fetal ECG monitoring research, there is the problem of finding adequate data for experiments. To ensure objectivity, the data should follow some standard. In conventional electrocardiography research, there are “gold standard” databases [82], which include large amounts of data and follow established criteria (such as the number of channels, electrode placement, sampling frequency, annotations, etc.). Nevertheless, there are no such databases in the case of fECG. There are some publicly available fECG data [82,83,84,85,86], but compared to the standardized ECG databases mentioned before, they are not sufficient. They include data from different stages of pregnancies, different electrode placements and fetal positions. Furthermore, pathological records are, understandably, almost totally nonexistent (as such data must be acquired during labor, and in the case of the risk of fetal hypoxia, this is impossible, as a cesarean section has to be performed and the pregnancy must be terminated).

For these reasons, like other authors [87,88], we arrived at the conclusion that to overcome the barriers in this line of research, it is essential to create a synthetic abdominal ECG signal generator that is able to simulate physiological, as well as pathological maternal (mECG) and fetal (fECG) signals, with the added capability to superimpose any type of noise on such signals [59,61]. Besides producing desirable synthetic signals [88,89,90] with realistic properties for our experiments, the generator enables us to evaluate the effectiveness and the quality of our optimized adaptive filters since it has the ability to produce the reference fECG signals, as well. As was mentioned above, using real fECG signals means they must be acquired invasively by placing the measuring electrode on the fetus’s scalp, which can only happen during labor. As was previously discussed, for an objective evaluation of the quality of signal filtering and the subsequent optimization of the deployed adaptive filters, the reference maternal and fetal signals are absolutely necessary. Thanks to our novel signal generator, it is now possible to test and evaluate the performance of adaptive filters during any stage of pregnancy since the generator allows the user to set the gestation age and adjust the desired signal properties (such as amplitude, frequency, etc.) based on this information.

## 3. Mathematical Description of LSM and RLS Algorithms

**Figure 1:**
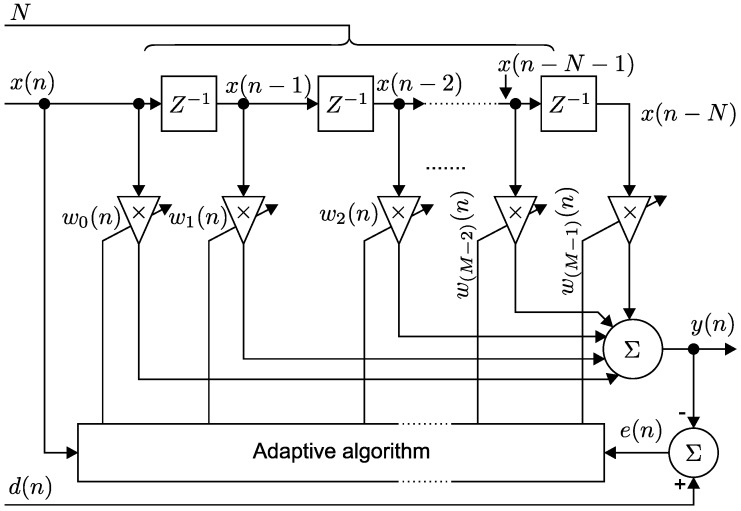
Block diagram for a linear *N*-th order adaptive Finite Impulse Response (FIR) filter with spinal structure.

In this section, we present a detailed mathematical description of the two main approaches used in our adaptive filters (see Figure 1): the stochastic gradient adaptation method, particularly the Least Mean Squares (LMS) algorithm (see [91,92,93]) and the optimal recursive adaptation method, particularly the Recursive Least Squares (RLS) algorithm [94,95,96].

Figure 1 shows the block diagram of a linear *N*-th order Finite Impulse Response (FIR) filter with a transversal structure (described in [97]).

In this figure, $\vec{x}\left(n\right)=\left[x\left(n-0\right),x\left(n-1\right),\ldots ,x\left(n-N\right)\right]$, is the input to the filter, $w_{i}$’s are the elements of the filter coefficient vector $\vec{w}=\left[w_{0},w_{1},\ldots ,w_{N}\right]$, which are in turn equal to the values of the impulse response $\vec{h}=\left[h_{0},h_{1},\ldots ,h_{N}\right]$, $z^{-1}$ represents the *z*-transform of the unit-step time delay, *N* is the filter order (or the often used filter length *M*, where N=M−1), d(n) is the desired (ideal) signal and y(n) is the (scalar) filter output.

### 3.1. Implementation of the LMS Algorithm

**Figure 2:**
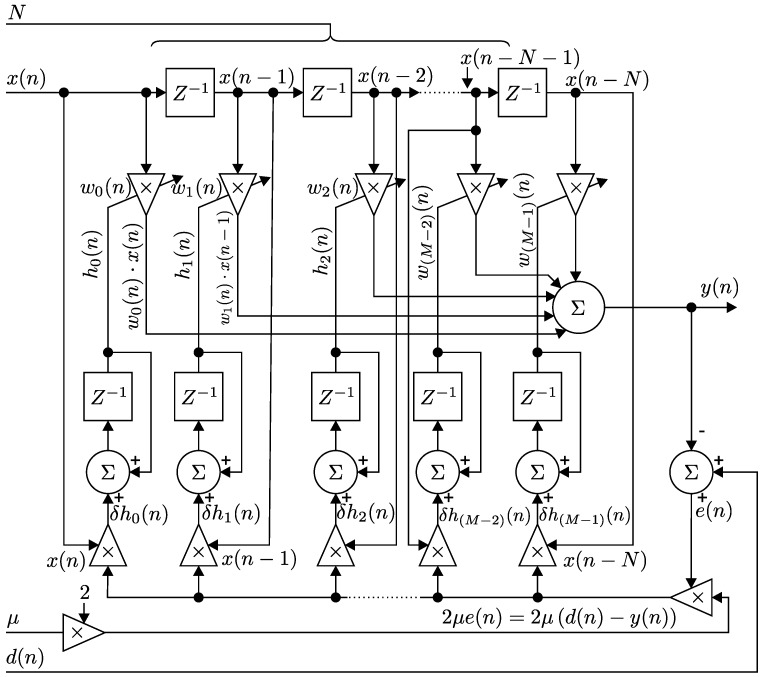
Block diagram of the adaptive FIR filter with spinal structure and the implemented *N*-th order LMS algorithm.

Figure 2 illustrates the block diagram of an adaptive FIR filter with spinal structure and the implemented *N*-th order LMS algorithm.

Each iteration of the LMS algorithm requires three different steps in the following order. First, the output of the FIR filter y(n) is calculated, according to Equation (1):

(1) $$y\left(n\right)={\vec{w}}^{T}\left(n\right)\cdot \vec{x}\left(n\right)=\sum_{i=0}^{N}w_{i}\left(n\right)\cdot x\left(n-i\right).$$

The symbol ${\left(.\right)}^{T}$ represents the transposition of the vector. FIR filters are defined by the property of individual coefficients $w_{i}$’s of their filter coefficient vector $\vec{w}$. $\vec{w}$ together with the filter order N=M−1 determines the performance of the designed FIR filter (for $M\in N^{+}$, $N\in N^{+},M>2$).

Subsequently, the value of the estimated error signal e(n) is given by Equation (2):

(2) e(n)=d(n)−y(n).

Finally, the values of the filter coefficient vector $\vec{w}\left(n\right)$ of the particular FIR filter are updated with respect to the next iteration according to Equation (3).

(3) $$\begin{array}{c} \vec{w}\left(n+1\right)=\vec{w}\left(n\right)+2\cdot \mu \cdot e\left(n\right)\cdot \vec{x}\left(n\right).\\ \vec{w}\left(n+1\right)=\vec{w}\left(n\right)+k_{\mu }\cdot e\left(n\right)\cdot \vec{x}\left(n\right).\\ \vec{w}\left(n+1\right)-\vec{w}\left(n\right)=2\cdot \mu \left[d(n)-y(n)\right]\cdot \vec{x}\left(n\right)=\delta \vec{h}\left(n\right)\;\forall n\in Z^{+}\\ \vec{h}\left(n+1\right)=\vec{h}\left(n\right)+\delta \vec{h}\left(n\right)\;\forall n\in Z^{+} \end{array}$$

Implementation of the LMS Algorithm in R is given by Equation (4) as follows:

(4) $$\begin{array}{cc} \mathrm{BEGIN} & \vec{w}\left(n=0\right)=\vec{0}\\ \mathrm{FOR} & (n=1,2,\cdots ,N):\\ & y\left(n\right)={\vec{w}}^{T}\left(n\right)\cdot \vec{x}\left(n\right)\\ & e(n)=d(n)-y(n)\\ & \vec{w}\left(n+1\right)=\vec{w}\left(n\right)+k_{\mu }\cdot e\left(n\right)\cdot \vec{x}\left(n\right). \end{array}$$

The step size (μ) plays a significant role in controlling the performance of the LMS algorithm. This parameter has a major impact on the speed and stability of convergence of the adaptive algorithm. Reaching the optimal value of μ (typically a small positive constant) is necessary for the correct functioning of the LMS algorithm, whereby:

- If the selected value μ is too small, the time required to find the optimal solution is too long.
- If the selected value μ is too large, the adaptive filter is unstable, and it will cause the deviation of the output.

### 3.2. Implementation of the RLS Algorithm

**Figure 3:**
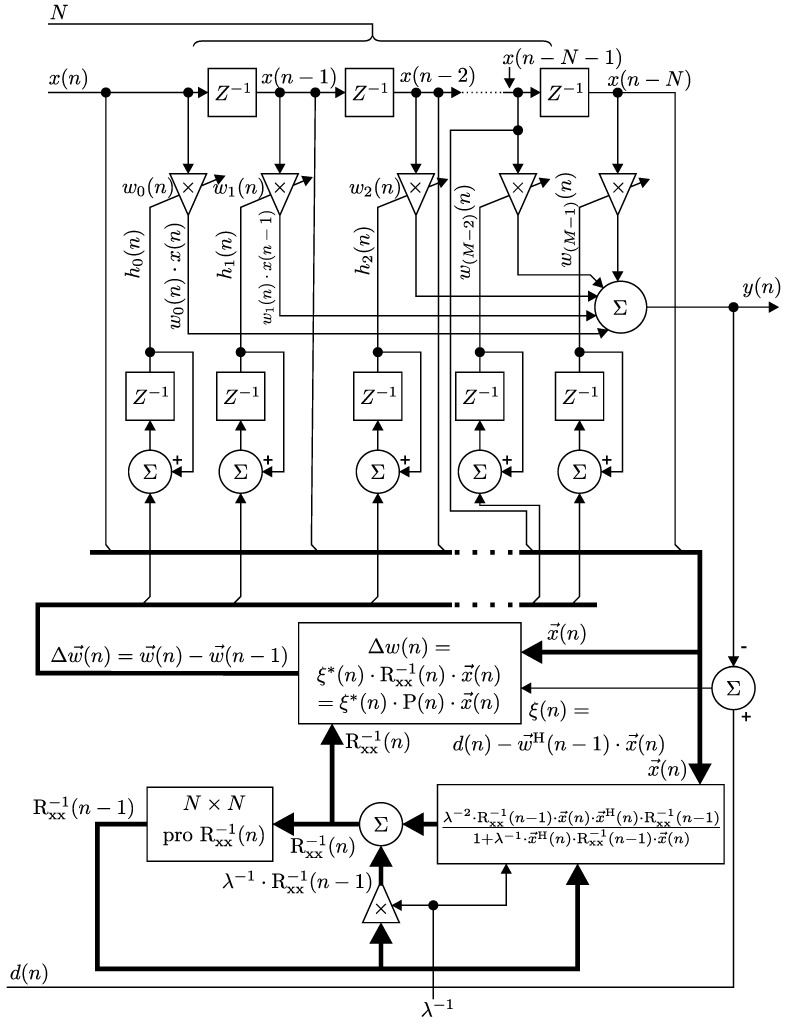
Block diagram of the *N*-th order RLS algorithm.

Figure 3 illustrates the block diagram of an adaptive FIR filter with spinal structure and implementation of the *N*-th order RLS algorithm.

Implementation of the RLS algorithm in R can be summarized as follows (Equation (5)):

(5) $$\begin{array}{cc} \mathrm{BEGIN} & \vec{w}\left(n=0\right)=\vec{0}\\ & P\left(n=0\right)=\delta^{-1}\cdot I\;\delta \in R\\ \mathrm{FOR} & (n=1,2,\cdots ,N):\\ & \vec{k}\left(n\right)=\frac{\lambda^{-1}\cdot P\left(n-1\right)\cdot \vec{x}\left(n\right)}{1+\lambda^{-1}\cdot {\vec{x}}^{T}\left(n\right)\cdot P\left(n-1\right)\cdot \vec{x}\left(n\right)}\\ & \xi \left(n\right)=d\left(n\right)-{\vec{w}}^{T}\left(n-1\right)\cdot \vec{x}\left(n\right)\\ & \vec{w}\left(n\right)=\vec{w}\left(n-1\right)+\xi \left(n\right)\cdot \vec{k}\left(n\right)\\ & P\left(n\right)=\lambda^{-1}\cdot P\left(n-1\right)-\lambda^{-1}\cdot \vec{k}\left(n\right)\cdot {\vec{x}}^{T}\left(n\right)\cdot P\left(n-1\right). \end{array}$$

P(n) designates the inverse correlation matrix of the input signal; $\vec{k}\left(n\right)$ is the gain vector; and Λ(n) is a diagonal matrix consisting of the weighting factors $\lambda^{n-i}$ (i.e., 1, λ, $\lambda^{2}$ …). The so-called “adaptation” or “forgetting factor” λ is in the range 0<λ<1 [91].

This parameter influences the process of “forgetting”, i.e., gives more weight to the recent samples of the error estimates compared with the older ones. If λ=1, then the estimation is without forgetting, i.e., equal to the ordinary method of least squares discussed in the previous section [96]. The weighting factor $\lambda^{n-i}$ influences the weights, where the input values are considered zero for i<1, and the last *n* samples are the most significant ones.

For implementation, the value of the “forgetting factor” is usually set in the range of 0.98<λ<1. A small value of λ causes the filter to place more emphasis on the recent samples of the observed data and tends to forget the past [78]. For i=n, i.e., $\lambda^{n-n}=\lambda^{0}=1$, the mathematical expression of the RLS algorithm is reduced to the expression for the LMS algorithm.

### 3.3. Comparison between the LMS and RLS Algorithms

The comparison between the proposed algorithms is performed below by using the cost function ξ and its minimization and by the estimation of the coefficient vector $\vec{w}\left(n\right)$ in R. The relationship between the coefficients $\vec{w}\left(n\right)$ and $\vec{w}\left(n\pm 1\right)$ is given by the properties of the implemented algorithm; see Table 1.

## 4. Methodology

Continuous signal processing of both abdominal (mECG + fECG) and thoracic (considered to be only mECG) signals may be a promising method for fetal ECG monitoring due to its technical feasibility and potential benefit in accurate diagnosis of fetal hypoxia. At the core of this signal processing challenge is the need for an adaptive system that allows extracting the desirable fECG component from the aECG signals (including mECG). Such filtered fECG signals can be then used for diagnostic purposes (including ST segment analysis, T/QRS rate determination and other morphological, as well as heart rate variability signal analysis).

### 4.1. A Multichannel Adaptive System

**Figure 4:**
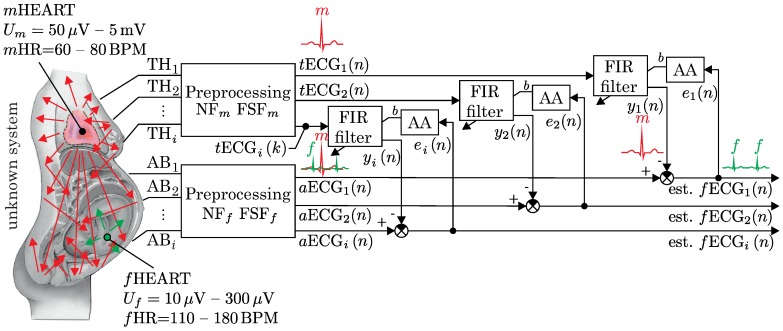
Theoretical multichannel adaptive system for fECG extraction.

A theoretical multichannel adaptive noise cancellation system is shown in Figure 4. It consists of two kinds of the input signals recorded from multiple leads: the abdominal ECG signals (${\mathrm{AB}}_{1}$–${\mathrm{AB}}_{n}$) and the thoracic ECG signals (${\mathrm{TH}}_{1}$–${\mathrm{TH}}_{n}$). Each abdominal signal is used as the primary input and consists of both maternal (mECG) and fetal (fECG) signals. The thoracic signal is considered to be composed of only the maternal component and is used as the reference input. The Finite Impulse Response (FIR) filter weights of the adaptive systems are updated by the training algorithms (Adaptive Algorithms (AA)) based on the back propagated error signal (*e*), which is equal to the desired fECG signal ($m{\mathrm{ECG}}_{1}$–$m{\mathrm{ECG}}_{n}$). The maternal component mECG is considered to be noise for elimination, and it is therefore subtracted from the abdominal ECG signal (aECG). After the subtraction step, the fECG signal with some noise is recovered based on the backpropagation training. The diagram includes the pre-processing part, which is not included in our research since the data that are used for the fECGextraction are recorded by the devices that involve analog pre-processing. These data are therefore already filtered by using, e.g., the Notch Filter (NF) and Frequency Selective Filters (FSF). The filters used for maternal and abdominal leads are different because the frequency range of the signals (fECG and mECG) slightly differs, as well. Therefore, you might observe different examples of Notch filters (${\mathrm{NF}}_{f}$, ${\mathrm{NF}}_{m}$) and frequency selective filters (${\mathrm{FSF}}_{f}$, ${\mathrm{FSF}}_{m}$) for fetal and maternal signals, respectively.

### 4.2. The Abdominal Maternal-Fetal Electrocardiogram Signal Generator

As mentioned earlier, the main aim of this paper is to confirm the theoretical assumption that the optimal settings of the adaptive algorithms used to filter aECG signals and extract high quality fECG signals from them depend on many variable factors (including the position of the primary and reference electrodes to acquire the input signals, the position of the fetus in the uterus, Gestational Age (GA), and others.)

To validate the above-mentioned assumptions, a number of experiments were performed for a specific combination of abdominal (AB) and thoracic (TH) electrodes positions. The main conceptual framework used in our system to generate non-invasive fECG and mECG synthetic signals is based on a dynamic cardiac dipole model for generating synthetic ECG signals developed by McSharry et al. [98] and further improved upon by Sameni et al. [88], as well as Behar [99]. A mixture of the maternal and fetal signals is generated by varying the size of the cardiac dipole and positioning it at different locations with respect to the maternal heart. The biopotentials (ECG signals) sensed by the body surface electrodes are linearly dependent on the cardiac dipole and a projection matrix that takes into account the evolution and the orientation of the cardiac dipole during the cardiac cycle. By superimposing two projections with different amplitudes, realistic fECG and mECG signal mixtures can be modeled.

#### 4.2.1. The Specifications of Our ECG Signal Generator

Surface electrodes sense the bioelectric (extracellular field) potentials that are generated by placing complex bio-sources inside a volume conductor (“a salt solution simulating the composition of body fluids”) and spread throughout this conductive medium. In fetal electrocardiography, these bio-sources can be categorized as cardiac sources (maternal and fetal heart) or noise sources (including muscular electrical activity generated by the movement or contractions of skeletal muscles: the EMG signal). Our novel ECG signal generator takes into account all of these signal sources as electric dipoles, which can change their position, magnitude and orientation. These dipoles are vectors represented by *x*, *y*, *z* in the Cartesian coordinate system, and their location together with electrode placements define the matrix of the signal propagation towards the significant points on the body surface.

Our ECG signal generator is designed to be very flexible, and it provides the options to set many relevant parameters as follows:

- Sampling frequency $f_{sr}=1/T_{\mathrm{sr}}$ in (Hz),
- Maternal heart rate, ${\mathrm{HR}}_{M}\equiv m\mathrm{HR}\in R^{+},30\leq m\mathrm{HR}\leq 200$ (bpm), fetal heart rate ${\mathrm{HR}}_{F}\equiv f\mathrm{HR}\in R^{+}$, 30 ≤mHR≤250 (bpm),
- Gestational age of the fetus GA in $R^{+}$, 20 ≤GA≤42 (weeks); GA reflects the amplitude and duration of the fECG elements, as well as the manual change of the fECG amplitude (generated by fHEART [A] and mECG mHEART [A]). The generator allows for the manual change of the length of the fECG signal elements. For more detail, please refer to [55],
- Heart’s positions including rotations along all axes: the position of the maternal heart (*M* = mother) in polar coordinates $M_{\mathrm{position}}=\left[r_{M},\phi_{M},z_{M}\right]$ and rotation of the maternal heart, i.e., rotation of vectorcardiogram (VCG) $M_{\mathrm{rotation}}=\left[\Psi_{M},\Theta_{M},\Phi_{M}\right]$, the position of the fetal heart (*F* = fetus) in polar coordinates $F_{\mathrm{position}}=\left[r_{F},\phi_{F},z_{F}\right]$ and rotation of the fetal heart $F_{\mathrm{rotation}}=\left[\Psi_{F},\Theta_{F},\Phi_{F}\right]$,
- Any number of chest electrodes (${\mathrm{TH}}_{i}$) and abdominal electrodes (${\mathrm{AB}}_{i}$).
- Position of the chest electrodes (TH) and abdominal electrodes (AB) in polar coordinates, i.e., ${\mathrm{TH}}_{i}\left[\rho ,\theta \;z\right]\equiv {\mathrm{TH}}_{i}\left[r,\phi \;z\right]$, for the experiments $i\in \langle 1,96\rangle$ and ${\mathrm{AB}}_{i}\left[\rho ,\theta \;z\right]\equiv {\mathrm{AB}}_{i}\left[r,\phi \;z\right]$, for the experiments $i\in \langle 97,168\rangle$,
- Modeling hypoxic conditions based on changes in T/QRS (i.e., hypoxemia, hypoxia and asphyxia) and in accordance with clinical guidelines for CTGand STANanalysis. The generator allows manual modeling of the hypoxic conditions; see [56],
- Modeling different types of noise and interferences (such as powerline interference, electromyographic (EMG) interference, baseline wandering, movement artifacts, and others), as well as amplitude, frequency and the position of the source of the interference in polar coordinates ϶=[A,f,r,ϕ,z].

**Figure 5:**
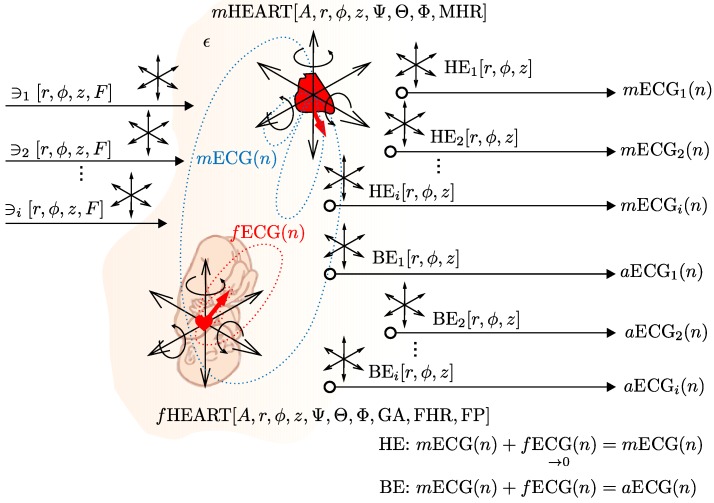
Parameter settings and specifications of our signal generator.

The above-mentioned specifications of the latest version of our ECG signal generator are conceptualized in Figure 5.

### 4.3. Data Selection Criteria

**Figure 6:**
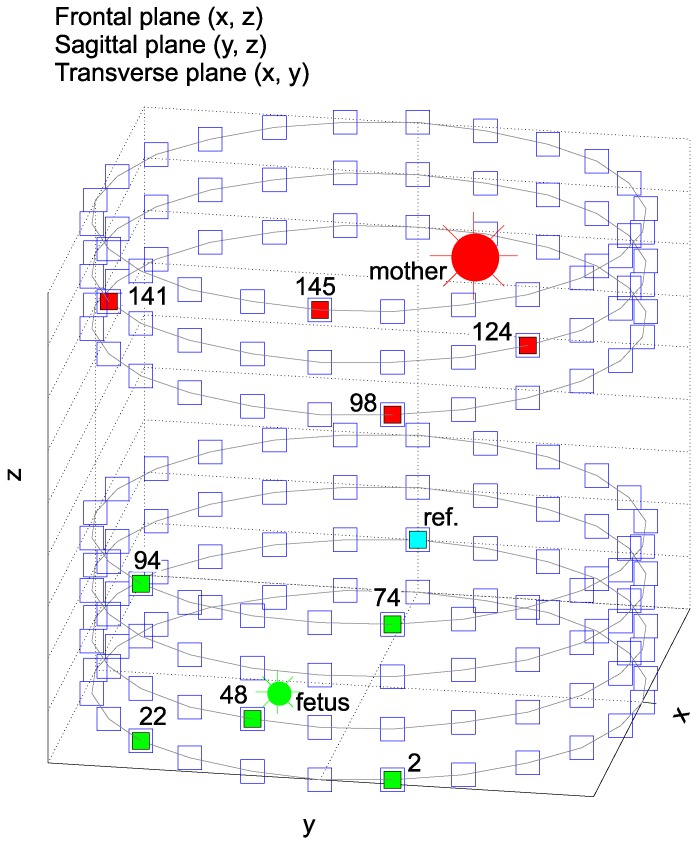
Abdominal (green) and chest electrodes (red) used for our experiments.

Figure 6 depicts a model of the volume conductor, the position of the maternal and fetal hearts, as well as the electrode placements. This model is based on the ECG signal generator designed by Sameni et al. [88] and Behar [99]. Our objective here is neither to propose nor develop a new model, but to modify the already existing models to serve our current requirements in the design, optimization and testing of the adaptive systems for fECG signal extraction. In Figure 6, the exact location of both maternal and fetal hearts and the corresponding positions of the electrodes in cylindrical coordinates can be seen. The model includes 168 electrodes, which are distributed around the volume conductor in two transversal planes representing the Thoracic (TH001–TH096) and abdominal (AE97-AE168) areas.

To capture all of the directions around both fetal and maternal hearts, we chose specific electrode positions for further experiments. This way, we could demonstrate that the optimal filter settings would change depending on electrode placement and as a result covered all maternal and fetal VCG directions. Additionally, the experimental electrode positions are also similar to those used by commercially available instruments used in fetal monitoring and fHR determination. The electrodes used for our experiments are labeled, and their colors correspond to the role they play in signal acquisition (blue for reference electrode, red for electrodes recording mECG from the thoracic area and green for recording both mECG and fECG from the abdominal areas).

### 4.4. Description of ECG Signals Used in Our Experiments

**Figure 7:**
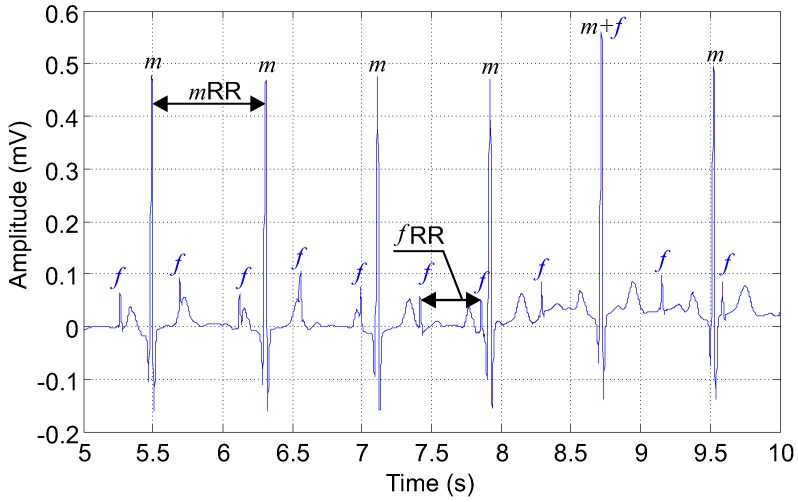
Primary input to the adaptive system (filter). A synthetic aECG signal generated by our signal generator and recorded by abdominal electrode AE002.

Here, we present examples of the input and output ECG signals used in our experiments. Figure 7 shows an example of an input signal fed to the adaptive system (filter). This ECG signal was recorded using the abdominal electrode AE002 (3M Corporate Headquarters, Maplewood, MN, USA) and contains both maternal and fetal components. Maternal and fetal R waves and the intervals between them (RR intervals, i.e., heart beats) are marked in the graph (*m*, maternal R wave; *f*, fetal R wave; mRR, maternal RR interval; fRR, fetal RR interval).

**Figure 8:**
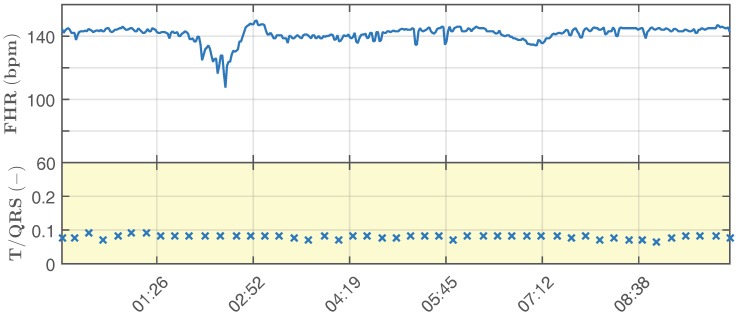
A sample simulation of physiological recording of the fHR and T/QRS.

The ECG signals used for our experiments are described as follows:

- Ideal mECG = reference signal for the adaptive system, i.e., TH98, TH124, TH141 and TH145 with a variable maternal heart rate (mHR) in the range of 65–85 bpm. This parameter takes into account the duration of mECG segments on mHR.
- Ideal physiological fECG signals: primary input to the adaptive system (abdominal electrodes AE2, AE22, AE48, AE74, AE94) with a variable fetal heart rate in the range of 110–150 bpm and T/QRS in the range of 0.05–0.1 (Figure 8).
- Ideal pathological fECG signal, which simulates fetal hypoxia (it is unstable and shows significant changes in the determined fHR and T/QRS).

The ECG signal parameters are described as follows:

- Length = 20 min, sampling frequency = 1 kHz, quantization step size = 0.1 mV. Please note that for clarity of the display, the recordings in the figures are 5 s long (Figure 7).
- Gestational age of the fetus = 40 weeks (this parameter affects the duration of individual fECG signal elements),
- Input Signal-to-Noise Ratio (SNR) for individual lead combinations,
- For our experiments, we used the head-down position known as the vertex presentation, which is the most probable (96.8%) and the appropriate presentation for birth. The presentation of the fetus is an important parameter since it influences the fetal cardiac signals recorded from the maternal body surface over different leads [27].
- The signal propagation model of the fECG and mECG signals, as well as the fetal and maternal heart positions were created based on information reported before [59,60,61,88].

**Figure 9:**
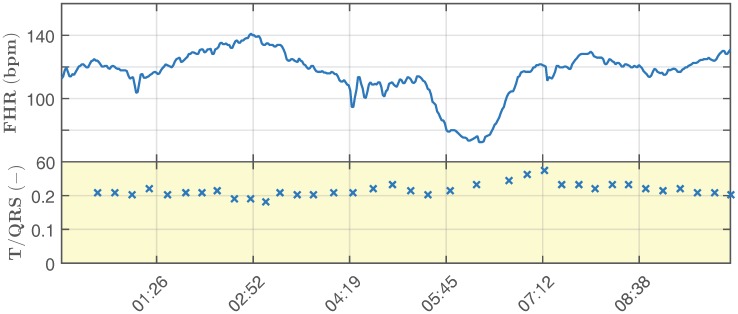
A sample simulation of pathological recording of the fHR and T/QRS.

Below, we describe the simulation of a real-life monitoring scenario, where a fetal hypoxia occurs. In a 20 min-long recording, the first half simulates the healthy state of the fetus, while the second half illustrates the changes that occur in the waveform as a consequence of the fetus suffering from hypoxia. Figure 8 and Figure 9 show the physiological and pathological parts of the recording, respectively. The figures include the calculated values of fHR (bpm) and T/QRS. This information corresponds to the visualization of the STanalysis by the Noeventa Medical ST analyzer (STAN S34). Please note that T/QRS [100,101] is calculated for 30 samples (for more information about T/QRS determination, please see [102,103]). The physiological fHR is relatively constant and fluctuates around the value of 140 bpm and the value of T/QRS oscillates around the value of 0.1. This fluctuation simulated the Beat-To-Beat (BTB) variability, which is physiological. In other works [57,58,104], the authors used data that were periodical, which did not correspond with the real-life attributes of fECG signals. The pathological fHR (Figure 9) oscillates in the range of 70–140 bpm, while the T/QRS can fluctuate around the value of 0.2 or exceed it significantly.

Our experiments showed that the type of data (physiological or pathological) did not have an impact on the performance of our adaptive systems (filters) reported here.

The model of hypoxia was used for the experiments; however, it did not influence the results at all. The result showed that the most significant factor that influences the quality of filtration is the value of SNR, which varies according to the electrode position and the filter settings. Since the information is almost the same, thus it would be redundant if presented, and the article would be even more comprehensive; the authors present only the results for the physiological data.

### 4.5. Evaluation of Signal Filtering Quality

The evaluation of the fECG signal extraction quality is based on the estimation of the similarity between the recovered fECG signals and the ideal (generated) fECG (reference) signals. Moreover, it is associated with the elimination (or at least reduction) of different types of noise. In our work, the parameters used to measure the efficacy of the fECG signal extraction were the Signal-to-Noise ratio (SNR), the Sensitivity ($S^{+}$) and the Positive Predictive Value (PPV), with the main emphasis on the SNR. These parameters are described in the section below [105].

#### 4.5.1. Signal-to-Noise Ratio

The relationship between the desired signal and the unwanted noise is quantified by the Signal-to-Noise Ratio (SNR) parameter. The calculation of this signal filtering quality measure is based on the calculation of its value before filtering (${\mathrm{SNR}}_{\mathrm{IN}}$) according to Equation (6) and after filtering (${\mathrm{SNR}}_{\mathrm{OUT}}$) by using Equation (7).

The value of ${\mathrm{SNR}}_{\mathrm{IN}}$ is defined as the following:

(6) $${\mathrm{SNR}}_{\mathrm{IN}}=10\cdot \log_{10}\left(\frac{\sum_{n=1}^{N}{\left\{{\mathrm{sig}}_{\mathrm{des}}\left(n\right)\right\}}^{2}}{\sum_{n=1}^{N}{\left\{{\mathrm{sig}}_{\mathrm{abd}}\left(n\right)-{\mathrm{sig}}_{\mathrm{des}}\left(n\right)\right\}}^{2}}\right)\;\left(\mathrm{dB}\right),$$

where ${\mathrm{sig}}_{\mathrm{des}}\left(n\right)$ denotes the desired fECG signal (i.e., the ideal waveform created by the synthetic signal generator) and ${\mathrm{sig}}_{\mathrm{abd}}\left(n\right)$ is the abdominal signal, which includes the unwanted signal considered as noise (i.e., the synthetic mECG signal after passing through the body of a pregnant woman, from her heart towards the abdominal electrodes). As the abdominal signal consists of the ideal fECG and mECG after passing through the unknown environment of the human body, it is necessary to subtract the desired signal from the noise in the denominator of Equation (7).

The value of ${\mathrm{SNR}}_{\mathrm{OUT}}$ is calculated as follows:

(7) $${\mathrm{SNR}}_{\mathrm{OUT}}=10\cdot \log_{10}\left(\frac{\sum_{n=1}^{N}{\left\{{\mathrm{sig}}_{\mathrm{des}}\left(n\right)\right\}}^{2}}{\sum_{n=1}^{N}{\left\{{\mathrm{sig}}_{\mathrm{rec}}\left(n\right)-{\mathrm{sig}}_{\mathrm{des}}\left(n\right)\right\}}^{2}}\right)\;\left(\mathrm{dB}\right),$$

where ${\mathrm{sig}}_{\mathrm{rec}}\left(n\right)$ denotes the recovered signal, i.e., the output of the implemented adaptive system (filter). In Equation (7), it is necessary to subtract the recovered signal from the noise because the aim is to determine only the noise contained in the fECG signal after adaptive filtering (system processing). The efficacy of this filtering operation can be determined by comparing the input and output values of the SNRs (i.e., ${\mathrm{SNR}}_{\mathrm{IN}}$ and ${\mathrm{SNR}}_{\mathrm{OUT}}$, respectively) [76,89].

When using real data, we face a problem, as we need to know the relative contributions of the fetal signal and noise to be able to calculate the SNRs. However, here, we could alleviate this problem by using synthetic data as this information is already available.

#### 4.5.2. Sensitivity

Sensitivity is a parameter that measures the proportion of positives that are correctly identified (e.g., the percentage of R or T waves). It is defined as shown in Equation (8):

(8) $$S^{+}\left[\%\right]=\frac{\mathrm{TP}}{\mathrm{TP}+\mathrm{FN}}\cdot 100,$$

where TP (True Positives) represents the number of correctly identified positives and FN (False Negatives) denotes the number of the significant points that were not detected. The detection of the R and T waves was performed by using ECG feature extractor included in the LabVIEW Biomedical TOOLKIT.

#### 4.5.3. Positive Predictive Value

PPV represents the proportion of TP results divided by the sum of true positive and False Positive (FP) results. It is defined by the following Equation (9):

(9) $$PPV\left[\%\right]=\frac{\mathrm{TP}}{\mathrm{TP}+\mathrm{FP}}\cdot 100,$$

In Equation (9), TP denotes the number of detected significant points of the signal (for example, R or T waves), and FP denotes the number of falsely-detected points. In the case of fECG signal extraction, the detection of FP occurs due to the maternal residual signal, which could not have been successfully filtered from the aECG signal. The calculations were based on the comparing of the reference (ideal) fECG signal and the estimated one. The detection was performed by using ECG Feature extractor included in the LabVIEW Biomedical TOOLKIT, it uses adaptive threshold, which adjusts according to the circumstances (signal properties). Other types of feature detectors, such as Pan–Tompkins’ algorithm adaptation to fetal R-peak identification, can be found in [106].

## 5. Results

In this section, we present our experimental results. Firstly, we describe the utility of optimization graphs as a novel evaluation technique for each particular electrode combination, then we summarize the optimal setting for all of the tested electrode combinations, and finally, we illustrate some examples of input and output signals for both adaptive filters (adaptive signal processing systems) based on the LMS and RLS algorithms, respectively.

### 5.1. Optimization Graphs

Here, we describe the utility of a unique method for the evaluation of the efficacy of bioelectrode placement on the quality of the fECG signal extraction using cost functions. We present 3D graphs that show the dependence of the ${\mathrm{SNR}}_{\mathrm{OUT}}$ on the step size μ and the filter order *N*. This approach helps us to find an operating area, where the adaptive filters produce their best possible results. With this approach, the optimal filter settings can be found for different electrode position combinations. In the following section, we present some examples for the 3D optimization graphs for the LMS and RLS algorithms that we used, whereas in Section 5.2, we summarize the rest of the tested electrode combinations.

#### 5.1.1. Cost Function for the LMS Algorithm

**Figure 10:**
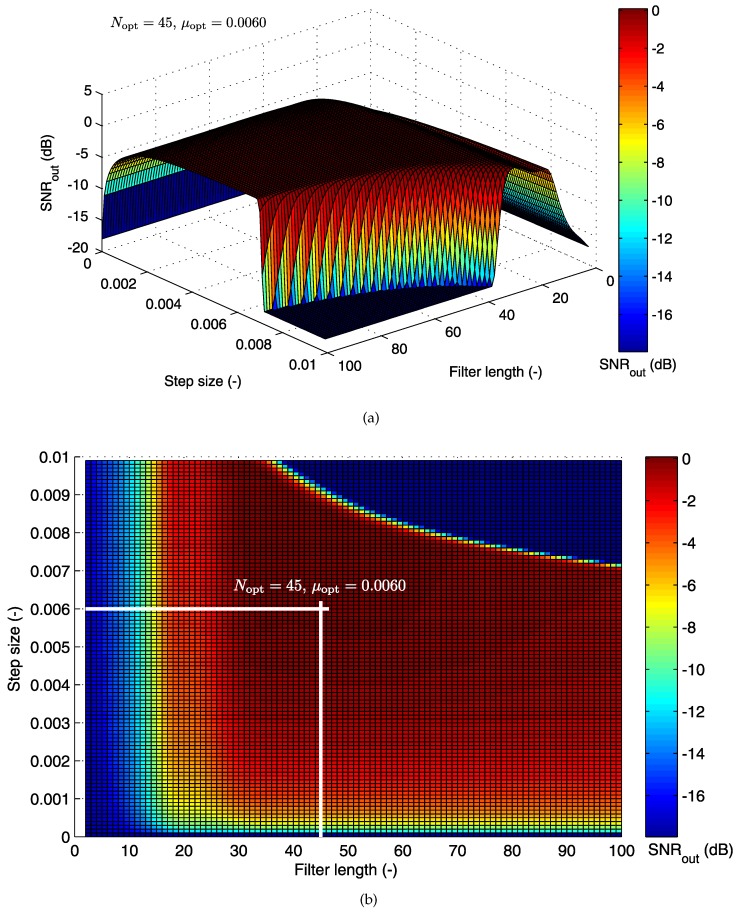
(**a**) 3D optimization graph for the LMS algorithm; (**b**) operating area with the optimal filter settings specified (for electrodes AE048-TE124).

**Figure 11:**
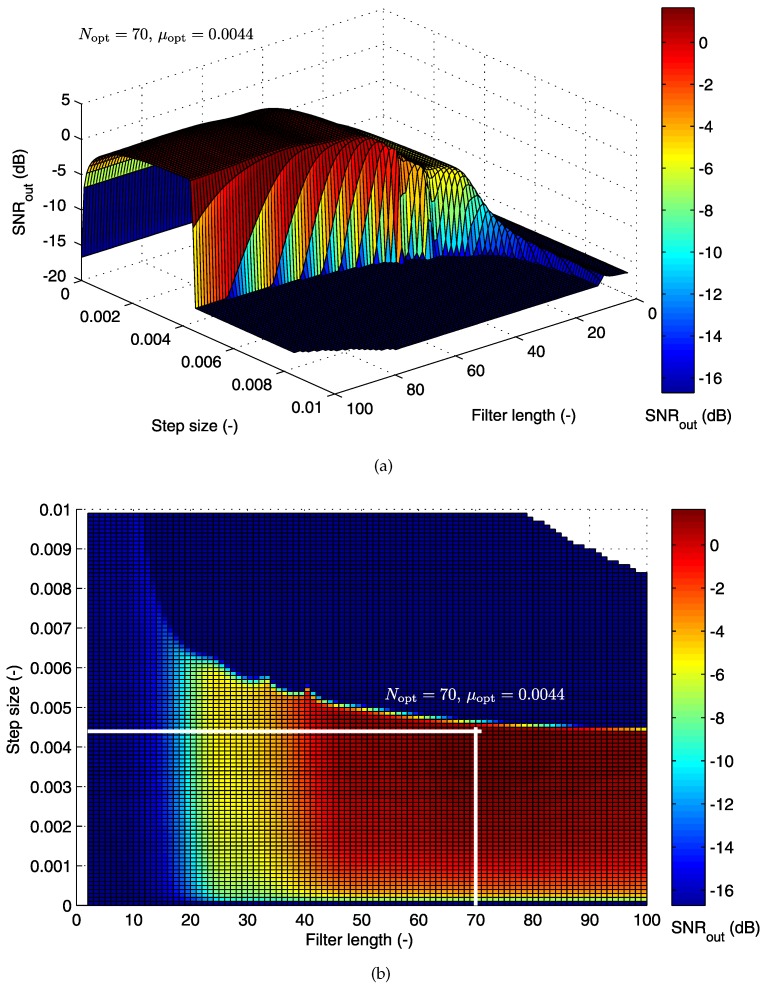
(**a**) 3D optimization graph for the LMS algorithm; (**b**) operating area with the optimal filter settings specified (electrode combination AE048-TE145).

For the LMS algorithm, the cost (loss) functions for different combinations of abdominal and thoracic electrode positions are plotted in Figure 10 and Figure 11. Figure 10a shows the 3D results for abdominal electrode AE048 and thoracic electrode TE124. This figure shows the quality of the adaptive filtering operation. Specifically, it shows the dependence of the ${\mathrm{SNR}}_{\mathrm{OUT}}$ as a function of changes in the values of *N* and μ. Additionally, Figure 10b shows the same information as in Figure 10a from a different perspective to illustrate the so-called operating area of the filter (the specific values of step size and filter order, which make the filter work most effectively).

For this given electrode combination, we can observe that the optimal value for the filter belongs to the interval N∈(30,70) and the step size μ∈(0.001,0.007). Outside of this defined area, there is a decline of signal filtering quality, and the adaptive system becomes unstable, especially for high values of both step size μ and filter order *N*, for which the decline is sharp. The best results for the LMS-based system were achieved at a global maximum of the depicted cost function ($N_{\mathrm{opt}}=45$ and $\mu_{\mathrm{opt}}=0.006$).

The higher the value of the filter length, the higher the computational cost. Based on Figure 10, we can say that for the AE048-TE124 electrode combination, it is not necessary to set N>50, as it has no positive effect on the value of ${\mathrm{SNR}}_{\mathrm{OUT}}$, nor consequently on the quality of signal filtering.

Another important consideration in the design of adaptive systems is the size of a convergence constant (step size) μ. It is an important parameter, which affects the stability of the system and its speed of convergence. Setting a high or a low value translates into producing two extreme results. The selection of a high value for the step size may result in obtaining a very fast optimal solution. However, in the case of the occurrence of a large error in the direction of the gradient, the estimation accuracy is reduced. A large step size is also related to the system’s instability and increases the estimation error in the subsequent steps (iterations). Choosing a small step size ensures high stability of convergence. At the same time, it slows the progress of the filtering action and thereby increases the inaccuracy of signal filtering in unsteady environments, as the ability of the system to adapt lags behind the properties of such environments. Based on these considerations, we carried out our experiments with μ∈[0.01–0.001].

According to the information in Figure 11, we can say that when using the combination of electrodes BE048-HE124, it is unnecessary to set μ<0.006. A smaller value of this step size does not produce a significant increase in ${\mathrm{SNR}}_{\mathrm{OUT}}$ (and thus, does not improve the quality of the filtering operation), but causes its gradual reduction.

Figure 11 also shows the optimization process for the AE048-TE145 electrode combination. For this combination, the primary electrode is the same as in the previous case; hence, the value of ${\mathrm{SNR}}_{\mathrm{OUT}}$ is the same as before. However, the reference electrode is changed, and therefore, it has a major impact on the LMS algorithm’s parameter settings.

The 3D graph in Figure 11a is very different from the 3D graph in Figure 10a. For this combination of electrodes, we observe that the main difference is in the larger range of values for the step size μ and filter order *N*. For these values, the adaptive system achieves quality signal filtering. The LMS algorithm operates optimally for lower values of μ and higher values of *N*. Figure 11b shows that the operating area is much smaller compared to the area specified for the previous combination of electrodes. Besides the objective area, a very steep decrease of the filtrating quality can be observed. The global maximum defined by the optimal values of tested parameters ($N_{\mathrm{opt}}=70$ and $\mu_{opt}=0.0044$) has moved towards higher values of the filter order, but lower values of the filter step size. The signal filtering quality (evaluated by the value of ${\mathrm{SNR}}_{\mathrm{OUT}}$) for this combination of electrodes is higher than that obtained in the previous case.

We can observe that for μ>0.0044, the value of the ${\mathrm{SNR}}_{\mathrm{OUT}}$ decreases significantly. Therefore, the optimum settings for this combination of electrodes should be chosen in the range μ∈[globalmaximum−0.001,globalmaximum] with step Δμ=0.0001. At the same time, the value of the filter length *N* should be higher than 60.

#### 5.1.2. Cost Function for the RLS Algorithm

In this section, we describe the 3D optimization graphs of the cost function for the second adaptive system that we tested by using the RLS algorithm, with two electrode combinations. In contrast to the LMS-based system discussed above, the second parameter of the RLS algorithm is called the “forgetting factor” λ (while N designates the filter length as before.) The “forgetting factor” falls in the range 0<λ<1. In practical applications, λ is usually a value close to one.

**Figure 12:**
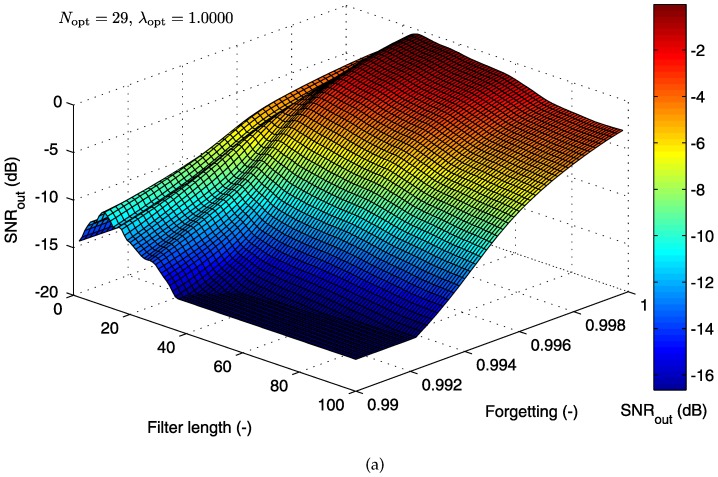

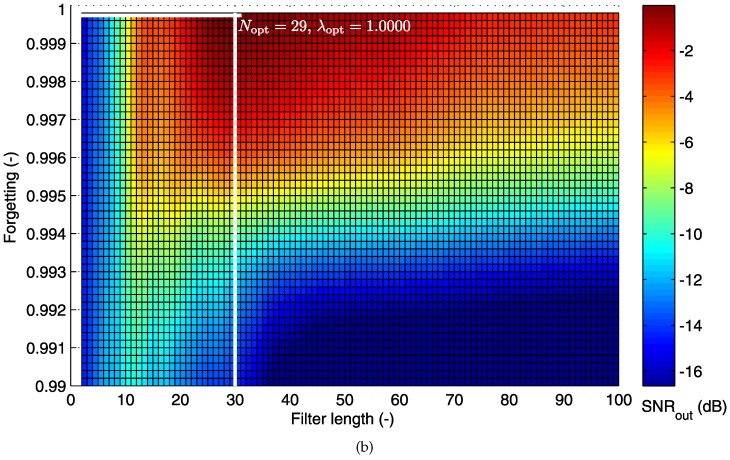
(**a**) 3D optimization graph for the RLS algorithm; (**b**) operating area with the optimal filter settings specified (electrode combination AE048-TE124).

Figure 12a shows the dependence of ${\mathrm{SNR}}_{\mathrm{OUT}}$ on the parameters λ and *N* for the combination of abdominal electrode AE048 and thoracic electrode TE124. The shape of the cost function graph for the LMS algorithm, presented in the previous section, differs significantly from the graph corresponding to the RLS-based system presented in this section. The signal filtering quality decreases gradually with lower values of the “forgetting factor”.

The operating area (see Figure 12b) is defined for high values of the “forgetting factor” and the values of N∈(25,65). The global maximum occurs at the optimal setting of $(N_{\mathrm{opt}}=29$ and $\lambda_{\mathrm{opt}}=1)$.

**Figure 13:**
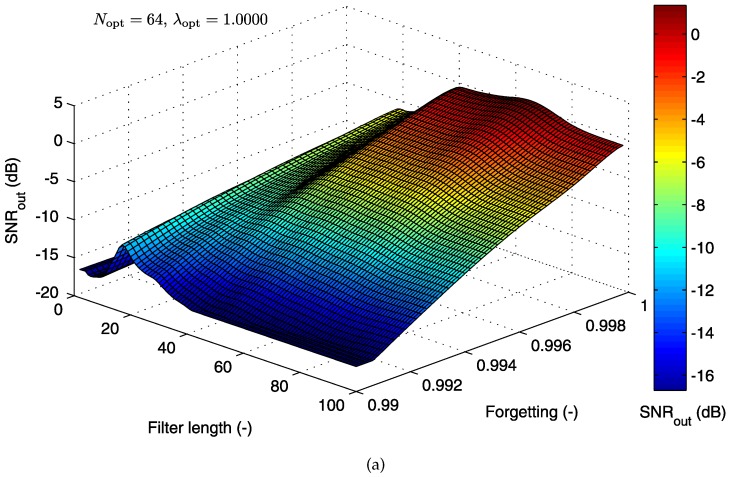

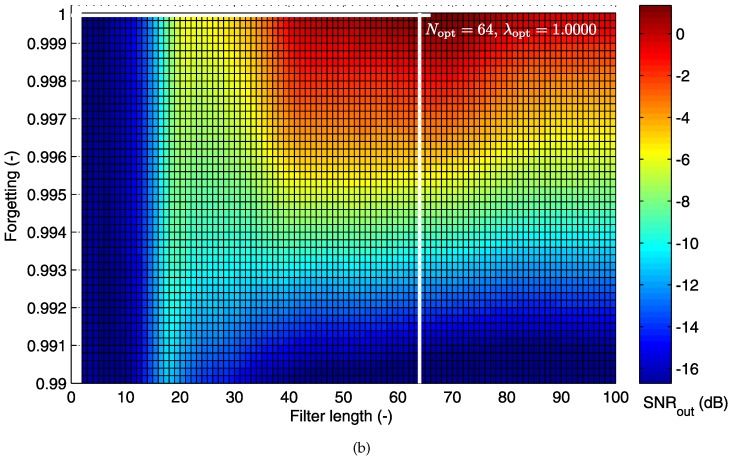
(**a**) 3D optimization graph for the RLS algorithm; (**b**) operating area with the optimal settings specified (for electrodes AE048-TE145).

Figure 13b shows the results for the combination of abdominal electrode AE048 and thoracic electrode TE145. The global maximum as defined by the optimal values of tested parameters ($N_{\mathrm{opt}}=64$ and $\lambda_{\mathrm{opt}}=1$) has moved towards higher values of the filter length, with lower values of the “forgetting factor.” The operating area moved in the same direction and changed its shape, as well (see Figure 13b). The signal filtering quality for this combination of electrodes is higher. The reason is that the thoracic electrode TE145 records the signal that is more similar to the abdominal maternal component, and hence, it is more suitable as the reference for its elimination.

The 3D optimization graphs clearly show that the RLS algorithm achieves better signal filtering results as λ increases, which is in agreement with the theoretical assumptions discussed in Section 3.2.

### 5.2. Electrode Placement-Based Optimization

The graphs in the previous section were examples of the optimization process. We tested the rest of the different electrode combinations in order to find the most suitable one for potential clinical application. This section summarizes our experimental results.

Table 2 and Table 3 show the results (with infant vertex presentation) using the LMS and RLS algorithms, respectively. The tables include values of input and output SNRs (${\mathrm{SNR}}_{\mathrm{IN}}$ and ${\mathrm{SNR}}_{\mathrm{OUT}}$) and the optimal settings (i.e., values of optimal filter length $N_{\mathrm{opt}}$ and step size $\mu_{\mathrm{opt}}$ for the LMS algorithm and the filter length $N_{\mathrm{opt}}$ and forgetting factor, for the RLS algorithm) corresponding to specific electrode combinations. Moreover, the tables include the values of sensitivity ($S_{\mathrm{QRS}}^{+}$ and $S_{T}^{+}$) and positive predictive value (${\mathrm{PPV}}_{\mathrm{QRS}}$ and ${\mathrm{PPV}}_{T}$), which show how successful the QRS detector was in distinguishing between the QRS and T complexes, respectively.

Based on our empirical experience and the outcomes of the implemented experiments, it can be stated that: whenever the mother and fetus are at rest (fHR→ constant, mHR→ constant, i.e., minimum muscle activity), the primary and reference signal changes are minimal (almost periodic signals), then λ=1. On the contrary, in the case of variable primary and reference signals (maternal movement, fetal movement, contractions, etc.), the “forgetting factor” will be λ<1. This fact agrees well with the theoretical assumptions, since the RLS algorithm is able to monitor the changes in the available to the variable Noninvasive (NI)-fECG and the mECG due to its ability to forget the parameters, in the case of λ<1. The effect of λ lies in the gradual forgetting of the older data, with the highest weight on the last measurement.

Theoretically, the “forgetting factor” should be defined as λ=1 to achieve a convergence of the parameters, but at the same time, the algorithm should be sensitive to changes in the parameters, i.e., the requirement that λ<1. Table 3 clearly shows that for some electrode combinations, the optimal value of the “forgetting factor” is λ=1, and for others, λ<1. Some of the values in the Table 2 and Table 3 are missing since the algorithms were not able to work properly due to high ${\mathrm{SNR}}_{\mathrm{IN}}$.

### 5.3. Examples of Filtered Signals

In this subsection, we present examples for the two adaptive system outputs that we designed and implemented. Our aim is to illustrate and summarize the impact of the electrode placement on the performance of these adaptive systems (filters) based on the LMS and RLS algorithms, respectively.

#### 5.3.1. The LMS-Based Adaptive System (Filter)

**Figure 14:**
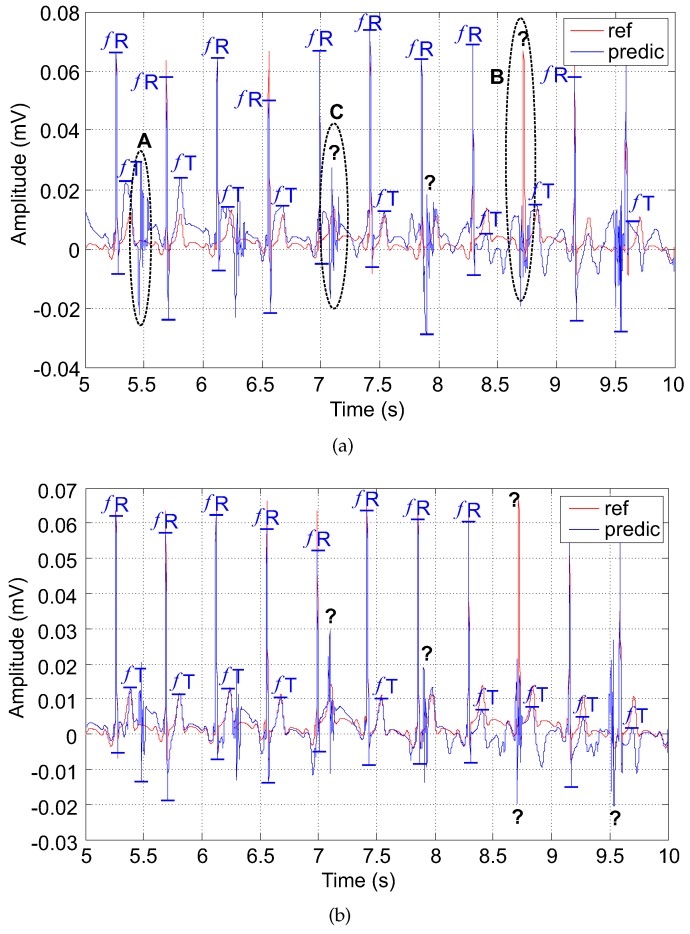
Output signals of the adaptive system (filter) using the LMS algorithm (**a**) electrode combination AE048-TE124; (**b**) electrode combination AE048-TE124.

**Figure 15:**
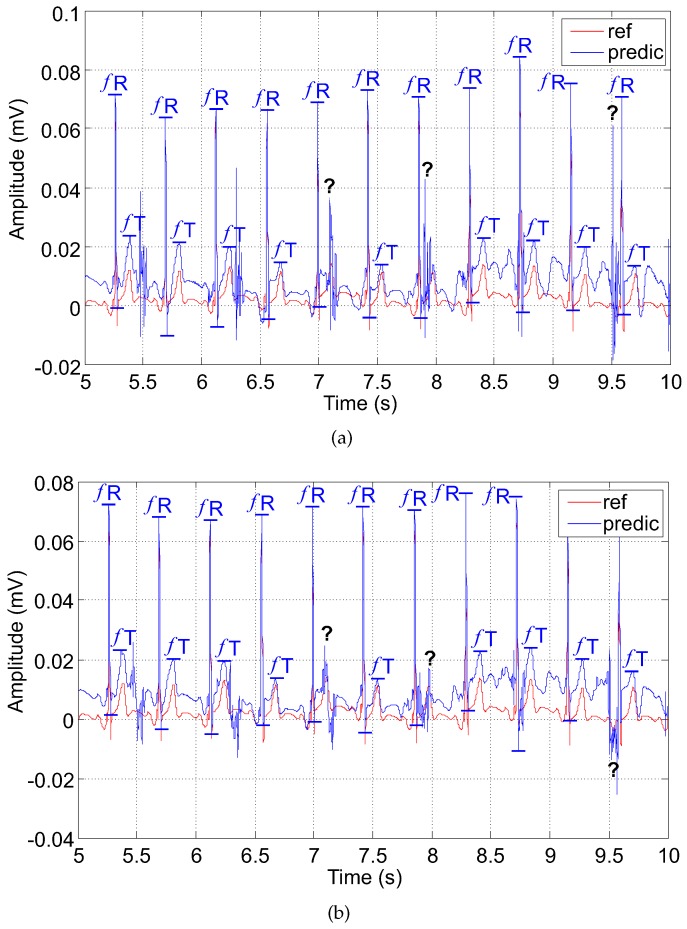
Output signal of the adaptive system (filter) using the RLS algorithm (**a**) electrode combination AE048-TE124; (**b**) electrode combination AE048-TE145.

Firstly, we introduce the fetal signals estimated by the LMS-based adaptive system. For the examples depicted in Figure 14 and Figure 15, the authors used the combination of electrodes AE048-TE145 since it has shown the best results according to Table 2 and Table 3. In Figure 14, the annotations A, B, C are used as follows:

- A is the maternal residue; it can be reversed with the fetal T wave due to its higher amplitude;
- B is the suppressed fetal R wave (fR); it may lead to false determination of fHR;
- C is the fetal T wave (fT) superimposed by the maternal residue; fT could not be detected.

To gain a clearer appreciation of each figure, the significant elements for the determination of fHR and T/QRS, i.e., fR and fT, are marked.

Generally, the LMS-based adaptive system tended to suppress some R-waves, and the maternal residue caused false detections of the fetal T wave. Such errors could cause either no detection due to the superimposition or detecting the residue as the T wave due to its high amplitude in both polarities (marked as question marks in Figure 14). Consequently, the value of the determined fHR was lower due to undetected R peaks.

#### 5.3.2. The RLS-Based Adaptive System (Filter)

In contrast, using the RLS-based system caused an elevation in the isoline; and the fHR determined from the predicted waveform was a little higher compared with the reference signal (see Figure 15). Determining the fT was more complicated due to the high maternal residue. On the other hand, the morphology of the estimated signal was comparable with the reference signal.

## 6. Discussion

In adaptive filter design and implementation, feasibility and computational cost are of paramount importance. Therefore, it becomes crucial to choose the filter settings (filter length, step size, “forgetting factor”, etc.) as a compromise between performance and hardware demands.

When the LMS algorithm was used, longer filter lengths (*N*) resulted in higher quality of signal filtering; however, this increased the computational cost. The convergence constant μ (step size) is an important parameter, which affects the stability of the system and its speed of convergence. Setting too high or low values for this parameter may cause system instability and an increase in the estimation error or a decrease in the algorithm’s speed: these changes may thereby increase the inaccuracy of the filtering process in unsteady environments.

Based on our empirical experience and the experimental results we obtained for the RLS algorithm, we can state: whenever both the mother and her fetus are at rest (fHR→ constant, mHR→ constant and therefore minimum muscle activity), the primary and reference signal changes are minimal (almost periodic signals), then λ=1. On the contrary, in the case of variable primary and reference signals (maternal movement and fetal movement in the womb, contractions, etc.), the “forgetting factor” will be λ<1. This fact agrees well with theoretical assumptions, since the RLS algorithm is able to monitor changes in the variable NI-fECG and mECG due to its ability to forget the parameters when λ<1.

The RLS algorithm produces better results than the LMS algorithm even though it causes the elevation of the isoelectric baseline. Its advantage is its accuracy, which, of course, is achieved at a higher computational cost. As high quality detection of fECG waveform morphology is the most important consideration in fECG signal processing, the RLS algorithm offers a better choice for effective fECG signal extraction. Its drawback, however, is that it overestimates the fHR value. In contrast, the LMS algorithm underestimates the fHR.

Our developed approach described above could be expanded and gainfully used to optimize other nonlinear signal processing systems, such as adaptive systems based on the Recursive Least Squares (RLS) algorithms, Adaptive Neuro-Fuzzy Inference Systems (ANFIS), and others [80,107]. The only difference in choosing other systems would be the selection of the parameters that need to be optimized. For example, when optimizing the RLS-based algorithms, we focused on the value of the “forgetting factor” and the filter length. For ANFIS, however, the most important parameters to set would be the shapes and number of membership functions, as well as the number of epochs [37,48,80].

In our work, we paid special attention to fECG signal extraction from abdominal ECG signals using adaptive filtering methods [77,78,79,80,81]. Our research demonstrates that the appropriate selection of optimal settings for adaptive systems (filters) offers the potential to significantly improve the diagnostic quality of the extracted fECG signals and consequently facilitate their clinical acceptance. Moreover, our proposed approach has the potential to emerge as a very useful complimentary method to support the conventional approaches currently used in the specialized medical device industry in the field of obstetrics and gynecology.

The drawback of our approach is the need for the reference mECG signal. This means using additional bioelectrodes and wires that might inconvenience the patient during labor and delivery. However, compared to other methods, our method improves the extraction of highly accurate fECG signals and enhances their clinical diagnostic quality. This in turn reduces the likelihood of distortion in these desirable signals. Therefore, our approach ultimately paves the way for more accurate detection and estimation of fetal hypoxic conditions in a noninvasive fashion.

Since the electrode placement and also the fetal heart position influences the fECG signal (fetal vectorcardiogram), it is theoretically possible to achieve the inverse information if the electrode placement is fixed and the fECG signal is available. This way, it would be possible to achieve the information about the fetal position even without utilization of the ultrasound; see [108,109,110].

In summary, a noninvasive, safe and cost-effective medical instrumentation system able to extract fetal ECG signals from abdominal signals would be a highly valuable tool in the timely detection and reliable diagnosis of fetal hypoxia during labor and delivery. The false positive diagnosis of fetal hypoxia because of inaccurate monitoring methods accounts for the currently large number of unnecessary caesarean sections performed. This number could be significantly reduced by our proposed method once it is statistically and clinically validated.

## 7. Conclusions

In this article, we presented a unique evaluation of adaptive filtering effectiveness in extracting fECG signals by using 3D optimization graphs of cost functions of the LMS and RLS algorithms for different combinations of abdominal and thoracic bioelectrode positions. This approach helped us to determine operating areas where the adaptive filters deliver their highest possible performance. We illustrated and described the influence of individual filter control parameters on the filtering performance in detail based on electrode combination placements. With this approach, the diagnostic contribution of the ECG signals in the maternal body during labor and delivery could be better understood, and the optimal filter settings for individual combination of abdominal and thoracic leads could be accurately identified.

As the optimal filter parameters depend on many different factors (electrode placement, fetal position, gestation age and others), they may vary from patient to patient and also for each individual patient during the pregnancy. Therefore, there is a need to automate the filter optimization process. Here, we introduced and described in detail a novel method for the optimization of adaptive filter control parameters where the main criterion for the selection of the optimal parameters (filter length, step size and “forgetting factor”) was the value of the SNR. The optimization was carried out for the LMS- and RLS-based adaptive systems (filters). However, our method could be extended and used for other adaptive systems and their associated parameters (for example, in ANFIS, where the number of membership functions and epochs could be optimized). Based on optimization outcomes, specific recommendations could be formulated and established to enable clinicians to achieve the highest possible fECG signal quality. This achievement in turn could lead to developing new diagnostic and detection methods for accurate determination of the occurrence of dangerous fetal hypoxic conditions during labor and delivery in a noninvasive and timely manner.

In summary, our results demonstrated that for effective fECG signal extraction, adaptive filtering performance depends on the maternal surface electrode placement. In other words, by choosing suitable electrode positions, the performance of adaptive filters (used to separate fECG signals from aECG signals) could be improved by means of optimizing their control parameters. The above results confirm that it is imperative to position the maternal electrodes while paying special attention to the direction of the maternal vectorcardiogram (mVCG), i.e., the fetal heart position. Because the filtering process impacts the morphology of the reference maternal signal, the thoracic leads have to be properly selected by considering the direction of the mVCG for filtering to be sufficiently effective. In our future research, we aim to obtain this reference signal from linearly independent abdominal leads to minimize the number of the required patient electrodes and wires. Moreover, we intend to use other datasets with different GA and to investigate thoroughly the influence of different fetal positions in addition to the most commonly-used fetal vertex presentation as discussed here.

## Figures and Tables

**Table 1:** Comparison between the LMS and RLS algorithms.

**LMS Algorithm**
$\xi_{\mathrm{LMS}}\left(n\right)=e\left(n\right)\cdot e\left(n\right)=e^{2}\left(n\right)={\left[d\left(n\right)-{\vec{w}}^{T}\left(n\right)\cdot \vec{x}\left(n\right)\right]}^{2}\to \min$
$\xi_{\mathrm{LMS}}\left(n\right)=d^{2}\left(n\right)-2\cdot {\vec{r}}_{dx}\left(n\right)\cdot \vec{w}\left(n\right)+{\vec{w}}^{T}\left(n\right)\cdot R_{xx}\left(n\right)\cdot \vec{w}\left(n\right)$
$\nabla \left\{\xi_{\mathrm{LMS}}\left(n\right)\right\}=\frac{\partial }{\partial \left\{\vec{w}\left(n\right)\right\}}\left\{d^{2}\left(n\right)-2\cdot {\vec{r}}_{dx}\left(n\right)\cdot \vec{w}\left(n\right)+{\vec{w}}^{T}\left(n\right)\cdot R_{xx}\left(n\right)\cdot \vec{w}\left(n\right)\right\}=\vec{0}$
$\nabla \left\{\xi_{\mathrm{LMS}}\left(n\right)\right\}=-2\cdot {\vec{r}}_{dx}\left(n\right)+2\cdot R_{xx}\left(n\right)\cdot \vec{w}\left(n\right)=\vec{0}$
$\vec{w}\left(n\right)=R_{xx}^{-1}\left(n\right)\cdot {\vec{r}}_{dx}\left(n\right)$
$\nabla \left\{\xi_{\mathrm{LMS}}\left(n\right)\right\}=-2\cdot e\left(n\right)\cdot \vec{x}\left(n\right)$
$\vec{w}\left(n+1\right)=\vec{w}\left(n\right)-\mu \cdot \nabla \left\{\xi_{\mathrm{LMS}}\left(n\right)\right\}=\vec{w}\left(n\right)+2\cdot \mu \cdot e\left(n\right)\cdot \vec{x}\left(n\right)$
**RLS Algorithm**
$\xi_{\mathrm{RLS}}\left(n\right)={\vec{e}}^{T}\left(n\right)\cdot \Lambda \left(n\right)\cdot \vec{e}\left(n\right)\to \min$
$\xi_{\mathrm{RLS}}\left(n\right)=\sum_{i=0}^{n}e_{i}\left(n\right)\cdot \lambda^{n-i}\cdot e_{i}\left(n\right)=\sum_{i=0}^{n}\lambda^{n-i}\cdot e_{i}^{2}\left(n\right)\to \min$
$\xi_{\mathrm{RLS}}\left(n\right)=\vec{d^{T}}\left(n\right)\cdot \Lambda \left(n\right)\cdot \vec{d}-2\cdot {\vec{r}}_{dx}\left(n\right)\cdot \vec{w}\left(n\right)+{\vec{w}}^{T}\left(n\right)\cdot R_{xx}\left(n\right)\cdot \vec{w}\left(n\right)$
$\nabla \left\{\xi_{\mathrm{RLS}}\left(n\right)\right\}=\frac{\partial }{\partial \left\{\vec{w}\left(n\right)\right\}}\left\{\vec{d^{T}}\left(n\right)\cdot \Lambda \left(n\right)\cdot \vec{d}\left(n\right)-2{\vec{r}}_{dx}\left(n\right)\cdot \vec{w}\left(n\right)+{\vec{w}}^{T}\left(n\right)\cdot R_{xx}\left(n\right)\cdot \vec{w}\left(n\right)\right\}=\vec{0}$
$\nabla \left\{\xi_{\mathrm{RLS}}\left(n\right)\right\}=-2\cdot {\vec{r}}_{dx}\left(n\right)+2\cdot R_{xx}\left(n\right)\cdot \vec{w}\left(n\right)=\vec{0}$
$\vec{w}\left(n\right)=R_{xx}^{-1}\left(n\right)\cdot {\vec{r}}_{dx}\left(n\right)$
$\vec{w}\left(n-1\right)=R_{xx}^{-1}\left(n-1\right)\cdot {\vec{r}}_{dx}\left(n-1\right)$
$\nabla \left\{\xi_{\mathrm{RLS}}\left(n\right)\right\}=-2\cdot e\left(n\right)\cdot \Lambda \left(n\right)\cdot \vec{x}\left(n\right)$
$\vec{w}\left(n+1\right)=\vec{w}\left(n\right)-\mu \cdot \nabla \left\{\xi_{\mathrm{RLS}}\left(n\right)\right\}=\vec{w}\left(n\right)+2\cdot \mu \cdot e\left(n\right)\cdot \Lambda \left(n\right)\cdot \vec{x}\left(n\right)$
$\vec{w}=\vec{w}\left(n-1\right)+\xi \left(n\right)\cdot \vec{k}\left(n\right)=\vec{w}\left(n-1\right)+\xi \left(n\right)\cdot P\left(n\right)\cdot \vec{x}\left(n\right)$

**Table 2:** Experimental results using the LMS algorithm for vertex presentation.

Electrodes	${\mathrm{SNR}}_{\mathrm{IN}}$	$N_{\mathrm{opt}}$	$\mu_{\mathrm{opt}}$	${\mathrm{SNR}}_{\mathrm{OUT}}$	$S_{\mathrm{QRS}}^{+}$	${\mathrm{PPV}}_{\mathrm{QRS}}$	$S_{T}^{+}$	${\mathrm{PPV}}_{T}$
	(dB)	(-)	(-)	(dB)	(%)	(%)	(%)	(%)
AE002-TE098	−24.94	15	0.0110	0.93	92.91	95.61	90.62	89.98
AE002-TE124	−24.94	57	0.0046	0.71	91.53	94.48	89.14	88.51
AE002-TE141	−24.94	25	0.0480	0.27	91.07	94.17	83.90	80.87
AE002-TE145	−24.94	22	0.0100	1.09	92.94	95.74	91.57	89.69
AE022-TE098	−21.73	21	0.0270	0.07	91.75	93.71	81.74	79.15
AE022-TE124	−21.73	39	0.0071	0.94	92.88	95.78	91.12	90.02
AE022-TE141	−21.73	23	0.0420	1.71	93.82	96.71	94.07	91.11
AE022-TE145	−21.73	19	0.0310	3.59	95.25	97.84	95.03	93.48
AE048-TE098	−17.09	17	0.0120	3.46	95.04	97.57	94.74	92.71
AE048-TE124	−17.09	45	0.0060	1.11	93.54	96.69	93.57	90.69
AE048-TE141	−17.09	21	0.0420	2.01	94.13	97.58	95.17	93.64
AE048-TE145	−17.09	70	0.0044	5.64	97.86	98.77	97.11	96.57
AE074-TE098	−26.36	21	0.0093	−0.97	91.81	94.09	—	—
AE074-TE124	−26.36	53	0.0035	−0.31	92.48	94.79	—	—
AE074-TE141	−26.36	87	0.0147	−3.45	—	—	—	—
AE074-TE145	−26.36	26	0.0097	−1.25	87.65	88.71	—	—
AE094-TE098	−31.71	19	0.0074	−2.85	—	—	—	—
AE094-TE124	−31.71	48	0.0034	−2.09	83.71	84.81	—	—
AE094-TE141	−31.71	27	0.0510	−3.93	—	—	—	—
AE094-TE145	−31.71	25	0.0121	−2.14	84.19	85.14	—	—

**Table 3:** Experimental results using the RLS algorithm for vertex presentation.

Electrodes	${\mathrm{SNR}}_{\mathrm{IN}}$	$N_{\mathrm{opt}}$	$\mu_{\mathrm{opt}}$	${\mathrm{SNR}}_{\mathrm{OUT}}$	$S_{\mathrm{QRS}}^{+}$	${\mathrm{PPV}}_{\mathrm{QRS}}$	$S_{T}^{+}$	${\mathrm{PPV}}_{T}$
	(dB)	(-)	(-)	(dB)	(%)	(%)	(%)	(%)
AE002-TE098	−24.94	11	1.0000	1.38	97.24	93.39	93.43	89.21
AE002-TE124	−24.94	51	0.9993	1.55	97.81	93.76	93.90	89.84
AE002-TE141	−24.94	37	0.9995	0.17	95.57	92.24	92.31	88.51
AE002-TE145	−24.94	31	0.9994	1.51	97.44	93.65	93.79	89.52
AE022-TE098	−21.73	17	1.0000	0.45	95.91	92.77	92.65	89.04
AE022-TE124	−21.73	39	1.0000	1.38	96.91	93.01	93.19	88.95
AE022-TE141	−21.73	24	1.0000	2.05	98.09	95.13	95.31	91.29
AE022-TE145	−21.73	15	1.0000	3.31	98.16	95.57	95.38	91.33
AE048-TE098	−17.09	13	1.0000	2.40	97.69	94.81	94.63	90.84
AE048-TE124	−17.09	29	1.0000	1.40	97.76	93.47	93.41	89.96
AE048-TE141	−17.09	26	1.0000	−0.09	94.21	91.86	80.74	78.06
AE048-TE145	−17.09	67	1.0000	5.30	98.75	98.31	97.46	95.79
AE074-TE098	−26.36	19	0.9997	−0.53	94.71	90.71	80.14	77.27
AE074-TE124	−26.36	75	1.0000	0.16	95.36	92.71	92.88	88.17
AE074-TE141	−26.36	41	0.9998	−5.97	—	—	—	—
AE074-TE145	−26.36	39	1.0000	−1.79	89.41	87.83	—	—
AE094-TE098	−31.71	23	0.9991	−5.07	—	—	—	—
AE094-TE124	−31.71	57	1.0000	−1.94	87.21	86.09	—	—
AE094-TE141	−31.71	29	1.0000	−5.32	—	—	—	—
AE094-TE145	−31.71	25	0.9993	−3.47	—	—	—	—
